# Controversial role of ILC3s in intestinal diseases: A novelty perspective on immunotherapy

**DOI:** 10.3389/fimmu.2023.1134636

**Published:** 2023-03-28

**Authors:** Yunshu Zhang, Xuefei Feng, Juan Chen, Jiahao Liu, Jianmin Wu, Hongpei Tan, Ze Mi, Pengfei Rong

**Affiliations:** ^1^ Department of Radiology, The Third Xiangya Hospital, Central South University, Changsha, Hunan, China; ^2^ Xiangya School of Medicine, Central South University, Changsha, China; ^3^ Department of Government & Public Administration, The Chinese University of Hong Kong, Hong Kong, Hong Kong SAR, China; ^4^ Key Laboratory of Biological Nanotechnology of National Health Commission, Xiangya Hospital, Central South University, Changsha, Hunan, China

**Keywords:** innate lymphocyte cell, plasticity, secondary lymphoid organ, tertiary lymphoid tissue, transcription factor, intestine homeostasis, inflammatory disease, cancer

## Abstract

ILC3s have been identified as crucial immune regulators that play a role in maintaining host homeostasis and modulating the antitumor response. Emerging evidence supports the idea that LTi cells play an important role in initiating lymphoid tissue development, while other ILC3s can promote host defense and orchestrate adaptive immunity, mainly through the secretion of specific cytokines and crosstalk with other immune cells or tissues. Additionally, dysregulation of ILC3-mediated overexpression of cytokines, changes in subset abundance, and conversion toward other ILC subsets are closely linked with the occurrence of tumors and inflammatory diseases. Regulation of ILC3 cytokines, ILC conversion and LTi-induced TLSs may be a novel strategy for treating tumors and intestinal or extraintestinal inflammatory diseases. Herein, we discuss the development of ILCs, the biology of ILC3s, ILC plasticity, the correlation of ILC3s and adaptive immunity, crosstalk with the intestinal microenvironment, controversial roles of ILC3s in intestinal diseases and potential applications for treatment.

## 1 Introduction

Innate lymphoid cells (ILCs) are lymphoid-derived innate cells that play a critical role in host defense and can be divided into the following four subsets: NK cells, ILC1s, ILC2s, and ILC3s (including lymphoid tissue inducer (LTi) or LTi-like cells) based on their functions, cytokine profile, and transcription factor (TF) expression during ILC development (1, 2).

ILC3s include three distinct lineages, NCR+ ILC3s (NKp46+ in mice and NKp44+ in humans), NCR- ILC3s and LTi/LTi-like cells; ILC3s primarily reside in the mucosa of the gastrointestinal tract, where they mediate the development of lymphoid tissue and mucosal protection (3–5). LTi cells can express the ILC3-specific TF RORγt and produce ILC3-specific cytokines, but they develop from different progenitor lymphatic tissue inducer precursors, while all other ILCs develop from precursors of innate lymphoid cells (6). LTi cells develop embryonically and initiate the formation of secondary lymphoid organs via LTβR signaling, while the development of LTi-like cells is postnatal and cannot induce the formation of secondary lymphoid organs (7). ILC3s are crucial in response to bacterial infection in the gut, especially for Citrobacter rodentium (8, 9). Once gut immune cells sense bacterial antigens, tissue-resident dendritic cells (DCs) and mononuclear cells produce numerous IL-23 and IL-1β-stimulating ILC3s to produce IL-17 and IL-22 to maintain intestinal homeostasis (4, 10–12). Furthermore, commensal flora influences the functional characteristics of intestinal NKp46+ cells. The levels of NKp46+ RORγt+ ILC3s are significantly decreased in germ-free mice, indicating that microenvironmental factors mediate these distinct effector cells in the gut, and commensal organisms influence gut immunity via a variety of sophisticated methods (13, 14). The development and functions of NCR+ ILC3s are largely dependent on RORγt and IL-7Rα (15–17). In addition, Notch acted on NCR- precursors. The Notch intracellular domain translocates into the nucleus, where it binds to recombining binding protein suppressor of hairless κ (RBP-Jκ), eliciting the expression of RORγt, AHR, and T-bet; thus, Notch is also an important signal for the generation of the NCR+ ILC3 population (18). ILC3s and LTi cells are the first line of defense in the response against pathogens. Thus, the deficit or overactivation of their functions will result in gastrointestinal disorders such as bacterial infection, inflammatory bowel disease (IBD), and colorectal cancer (CRC) (19).

Recent studies have found that ILC3s and LTi cells are emerging as an essential innate lymphocyte population for intestinal infection and respond distinctly to different intestinal diseases (20). The balance of ILC3s and LTi cells ensures host homeostasis, and their regulation may contribute to the alleviation of both tumor and inflammatory diseases. Here, we focus on recent encouraging findings in the field of ILC3s and highlight the biological mechanisms of ILC3s in intestinal diseases.

## 2 Main transcription factors mediating ILC3s and LTi/LTi-like cells

### 2.1 AHR

ILC3s express the specific TF aryl hydrocarbon receptor (AHR) (16, 21). AHR is a cytosolic sensor of small polycyclic aromatic compounds and can regulate Notch signaling in the formation of isolated lymphoid follicles (ILFs). It has been observed that AHR overexpression in NK cells could increase Notch2 expression, which suggests that AHR could modulate the Notch pathway (22, 23). TCDD, an AHR ligand, has also been found to induce the expression of Notch transcripts in the gut through an AHR-dependent pathway. AHR-/- mice exhibit significant RAR-related orphan receptor γt+ (RORγt) ILC deficiency, leading to a decrease in IL-22 production and poor protection against intestinal bacterial infections (21). Similarly, in a mouse model that lacks the expression of RBP-Jk, which regulates the output of Notch signaling, the frequency of RORγt+ ILCs was notably reduced, supporting the idea that Notch signaling regulates RORγt+ ILCs. Interestingly, increased apoptosis in RORγt+ ILCs is observed in the intestine of adult AHR-/- mice but not in that of AHR-/- fetal mice (21).

Similarly, AHR-/- mice lack postnatally imprinted Cryptopatche (CP) and ILFs but not embryonically imprinted Peyer’s Patche (PP), indicating that RORγt+ LTi cells play an important role in the generation of postnatal intestinal lymphoid tissues and that heterogeneity in cell types plays a role in the organogenesis of lymphoid tissue. The effects of AHR in inducing lymphoid organogenesis are supposed to be related to its role in facilitating LTi cell development, as LTi cells can recruit stromal cells and other lymphoid cells to form the lymphoid structure. Moreover, the receptor tyrosine kinase c-kit has also been identified as a downstream target of AHR. AHR interacts with the canonical xenobiotic responsive (XRE) element on c-kit, inducing the transcription of c-kit. Recent studies have proven that c-kit can also regulate the frequency of postnatal intestinal RORγt+ ILCs and lymphoid organogenesis. In mice that express a receptor with impaired kinase activity, RORγt+ ILC frequency diminished considerably, and the number of CPs and ILFs decreased as well (24). These results suggest that AHR is important for the maintenance and activity of postnatal RORγt-ILCs, thereby mediating lymphoid organogenesis.

In addition, AHR activated by its ligands may result in Ncr1 fate-mapped ILC3s expressing higher levels of CD117 and IL-22, leading to better protection against pathogens (25). Compared to wild-type ILCs, AHR-deficient ILCs produce less IL-22, which is closely associated with a high bacteria load (26). It has also been found that AHR ligands that drive ILC3s are mostly endogenous ligands, such as Kynurenine (tryptophan catabolite), and not natural diet ligands (21, 27). These results suggest that intestinal commensal bacteria that participate in the synthesis of endogenous AHR ligands could play an important role in the AHR-Notch pathway and influence the generation of ILCs and postnatal lymphoid tissue.

### 2.2 ROR

RORγt, with 495 amino acids encoded by the Rorc gene (28), is mainly expressed in TH17 cells and thymocytes (28). RORγt is the key TF for ILC3s and LTi cells, regulating their development (29). The formation of ILC3s in RORγt-knockout mice was completely suspended, suggesting the paramount role of RORγt in ILC3 formation, but the precise regulatory mechanisms are still unclear (30). Recent studies have shown that ILCs do not require RORγt and RORα, another crucial ROR TF, for survival, but the continuous expression of RORγt and RORα is closely associated with their metabolism, proliferation and functions (31). When RORγt and RORα expression is lacking, the expression of crucial metabolism regulators such as Arg-1 diminishes notably, and thus, the cells lose their phenotype and functions. Meanwhile, RORγt- RORα- T-bet+ NCR+ ILC3s also convert to ILC1s (31). The expression of RORγt is a potential signature to recognize IL-17- and IL-22-producing cells in both adaptive and innate immune responses. RORγt is crucial in controlling ILC3s producing IL-17 and IL-22 and promoting the development of LTi cells (12). LTi cells constitutively express RORγt, and IL-23 can upregulate the expression of RORγt (12, 21). In addition, current studies have confirmed that one of the most important functions of RORγt is mediating the differentiation of proinflammatory TH17 cells (28, 32–34). Thus, RORγt is a promising therapeutic target in treating gut inflammation and chronic autoimmune diseases (34). In the context of temporary intestinal infection with Citrobacter rodentium, inhibition of RORγt in mice reduced cytokine production from TH17 cells but not ILCs, selectively preserving innate immunity (34). Withers et al. found that the transient inhibition of RORγt led to remarkably favorable results in mouse models of intestinal inflammation and suggested that the inhibition of RORγt is an effective strategy during intestinal inflammation. It has been identified that ILCs expressing RORγt, Thy1 and stem cell antigen 1 (SCA-1) accumulate in the inflamed gut, which is triggered by IL-23, but RORγt- mice fail to develop innate colitis under IL-23 stimulation. Thus, RORγt, a TF of IL-23, has a functional role in IL-23-induced innate colitis (35).

### 2.3 T-bet

T-bet inhibits the transcription of Rorc, downregulating the expression of RORγt (36). It has been confirmed that ex-ILC3s, ILC3-to-ILC1 transitional subsets, upregulate the expression of T-bet, which indicates that the balance between RORγt and T-bet plays an important role in ILC3-ILC1 equilibrium. Fiancette et al. observed that T-bet-/- RORγt-/- ILC3s failed to convert to ILC1s, but a group of ILCs with unknown functions. They also failed to produce IL-22 in response to IL-23 (31). This finding suggests that in addition to RORγt, T-bet is indispensable for ILC3-to-ILC1 conversion. Similarly, Stehle et al. found that T-bet deficiency could reverse the suppressive effect on lymphoid organogenesis caused by RORγt deficiency in mouse model, and T-bet-RORγt- innate lymphoid progenitors (ILCPs), instead of ILC3s, restored the intestinal barrier via the secretion of IL-22 (37). Thus, the low expression of T-bet in ILC3s ensures the equilibrium of ILC subgroups and the homeostasis of host immunity.

## 3 Connections and reversible plasticity between ILC3s and other subtypes of ILCs

**Figure 1:**
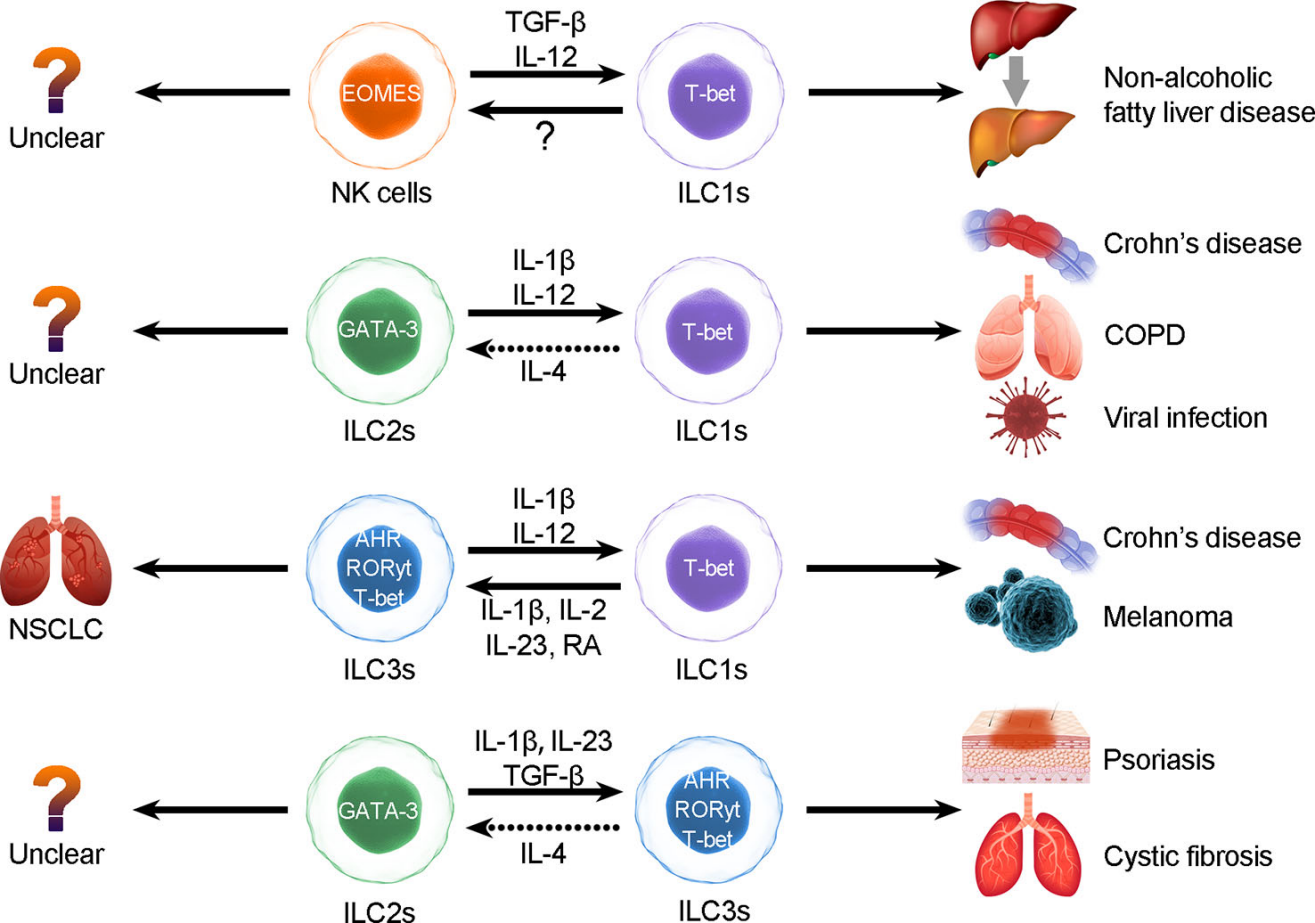
ILC plasticity. ILCs can be converted to each other through the induction of cytokines, but the interconversion may induce diseases. The types of ILC conversion are divided into the following four types: 1) conversion between NK cells and ILC1s; 2) conversion between ILC2s and ILC1s; 3) conversion between ILC3s and ILC1s; and 4) conversion between ILC2s and ILC3s.

ILCPs can differentiate into ILC1, ILC2 and ILC3 subtypes, and the development of those ILCs depends on TFs, such as RORγt, T-bet and GATA3. All ILC subgroups reserve other lineage-specific genes and can be activated under stimulation. The activation of ILC subset-associated signature cytokine loci allows ILC plasticity and expression of specific cytokines that were initially expressed by other ILC subgroups (38) (**Figure 1**).

### 3.1 Conversion between ILC1s and ILC3s

In the presence of IL-23, IL-12 and IL-1β, which are derived from tissue-resident myeloid cells (3), CD127+ ILC1s differentiate into ILC3s in a manner dependent on the TF RORγt. This process can be accelerated in the presence of retinoic acid (RA) secreted by DCs (30, 39). In contrast, DC-derived IL-12 could promote ILC3 differentiation to CD127+ ILC1 cells in vitro (39). Reversible conversion between CD127+ ILC1s and ILC3s is a process reliant on RORγt, RORα, T-bet, and other cytokines they are exposed to (31). In response to bacterial infections or IL-1β and IL-12 (40), NCR+ ILC3s convert into IFNγ-producing ILC1s, following RORγt downregulation and upregulation of T-bet (39). Similarly, Muraoka et al. found that C. jejuni infection-induced IL-12, IL-15, and IL-18 could upregulate T-bet expression and downregulate RORγt expression and therefore promote ILC3-to-ILC1 conversion and inflammation progression. In addition, hypoxia-inducible transcription factor (HIF) 1α could upregulate the expression of T-bet and support the conversion to ILC1s (41). It has also been observed that ILC3s isolated from RORα-/- RORγt-/- mice acquire more potent ILC1 types than those isolated from RORγt-/- mice. T-bet-associated regulon activity was particularly predicted in RORα-/- RORγt-/- ILC3s, while it was predicted in only one cluster of RORγt-/- ILC3s (31). These results suggest that RORγt deficiency initiates ILC3-to-ILC1 conversion and that T-bet-associated regulon activity is fully activated in the absence of RORα, which fully realizes conversion toward the ILC1 phenotype.

### 3.2 Conversion between ILC2s and ILC3s

Recent studies identified that the Notch transcriptional complex binds to the Rorc gene locus and promotes RORγt expression, conferring ILC3-like functions to ILC2s (42). Notch-induced ILC2s can produce IL-17 (ILC3-characteristic cytokine) through Gata3 expression and increased RORγt expression (42). Therefore, Notch may be a crucial driver in triggering ILC conversion. TGF-β signaling also elicits ILC2s to differentiate into IL-17-producing ILC3-like cells following the expression of IL-23R (43, 44). Ligands of Toll-like receptor 2 activate ILC3s to produce IL-5 and IL-13, which indicates that ILC3s could convert into ILC2s and that the conversion between ILC3s and ILC2s is bidirectional (44).

### 3.3 Conversion between ILC2s and ILC1s

Under homeostatic conditions, the key TF GATA3 binds to the Ifng-controlling element and restricts IFN-γ production and ILC2-to-ILC1 conversion (45). ILC2s can also differentiate into ILC1s. Under the stimulation of IL-1β and IL-12, ILC2s enhanced the expression of ILC1-related genes and acquired an ILC1 phenotype with decreased expression of the TF GATA-3 (46). IL-1β could induce this process. In contrast, only IL-12 stimulation could not lead to conversion, which suggests that IL-12 may regulate downstream signaling of the conversion process. IL-1β induces IL-12 receptor B2 and upregulates the expression of ST2 and IL-17 receptor B, which are IL-33 and IL-25 receptor components on ILC2s (45). Exposure to IL-4 could reverse this process (47). However, IL-4 could not induce ILC1-to-ILC2 conversion. ILC2s downregulate the expression of ILC2-specific GATA-3 and IL-33R (ST3) in the presence of cigarette smoke and are gradually converted into IFN-γ-producing IL-12R+ IL-18R+ ILC1s (44). The conversion of ILC2s to ILC1-like cells can be reversed by IL-4; however, it has not been proven that ILC1s can convert to ILC2s (43, 44).

### 3.4 Conversion between NK cells and ILC1s

It has been observed that NK-to-ILC1 conversion could be induced by TGF-β and IL-12 in a mouse model, while subsets with mixed NK and ILC1 features have been discovered in humans (48). The TF TGF-β supports NK-to-ILC1 conversion by upregulating the expression of T-bet while repressing EOMES. Another TF, SMAD4, binds to TGF-β receptor 1, suppressing the TGF-β signaling pathway. In the SMAD4-deficient mouse model, NK cells respond more strongly to TGF-β than those in normal mice, and the abundance of ILC1s is higher than that in the control group (49). However, the ILC1-to-NK conversion is still unclear.

In short, we suggest that the plasticity of ILCs is closely related to transcriptional regulation and that conversions are highly related to distinct pathological processes (50). In intestinal diseases (40), IL-12 elicits ILC1s to switch into ILC3s, whereas IL-1 plus IL-12 collaboratively induces ILC2s to convert into ILC1s in respiratory diseases, and overactivation of the IL-17 and IL-22 pathways results in escalation of NCR+ ILC3s in patients with psoriasis (51–53). In patients with COPD, the increase in ILC1s appears to be associated with poor prognosis (44). Blockade of conversion-promoting cytokines such as IL-1β and IL-12 or activation of IL-4 may help to reverse ILC2-to-ILC1 conversion and alleviate chronic inflammation. Thus, converting ILCs may result in autoimmunity, inflammation, and carcinoma (42). Therefore, further exploration of conversion between ILCs is necessary, as the conversions between distinct ILC subgroups may be useful biomarkers or predictable signs (**Figure 1**).

## 4 ILC3s in initiating the secondary lymphoid organ

**Figure 2:**
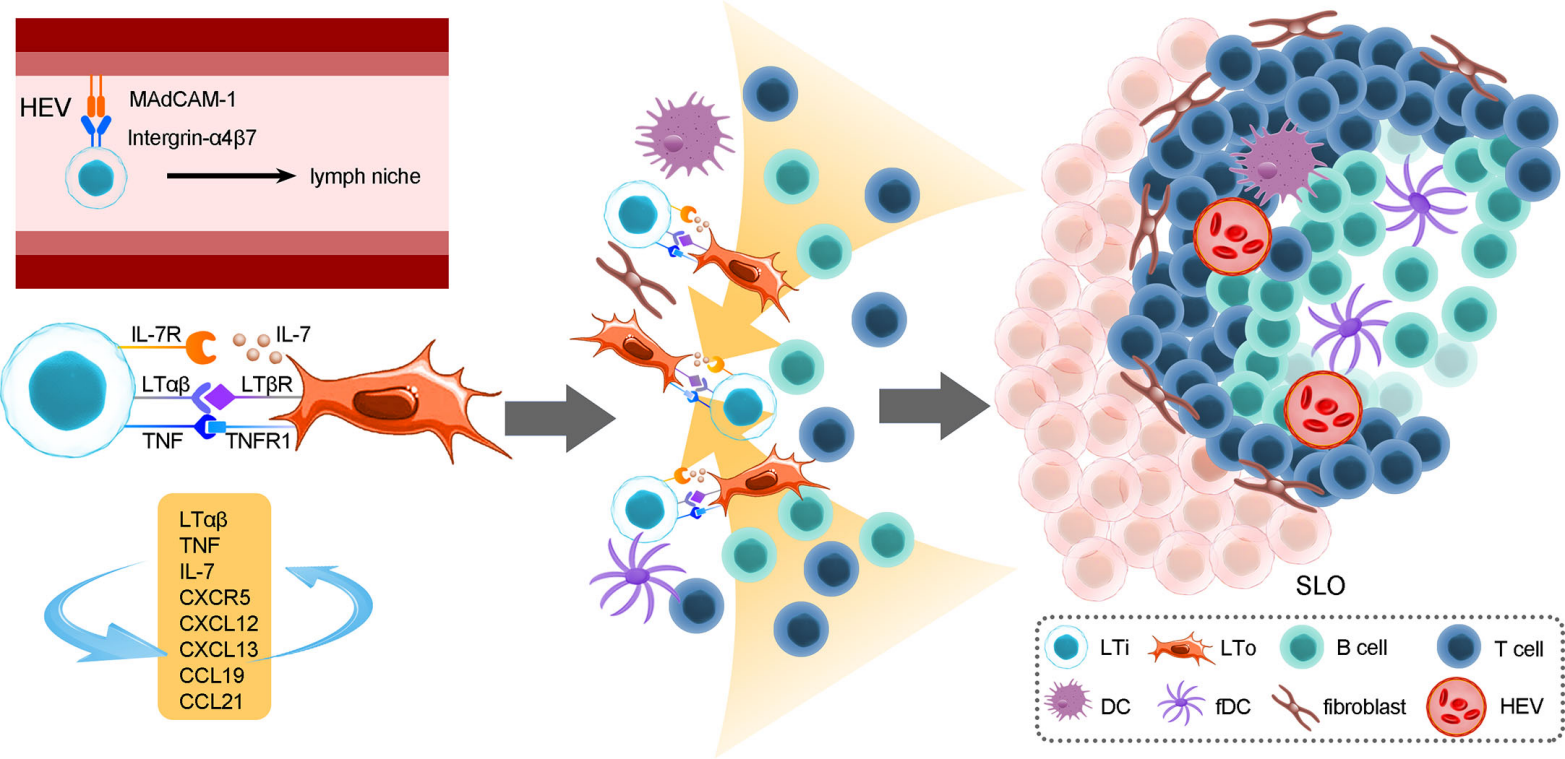
The roles of LTi cells in the development of secondary lymphoid organs. The formation of SLOs in the mouse intestine strictly depends on LTi cells. SLO development is highly reliant on the crosstalk between LTi cells and LTo cells and consequently immune cell recruitment, reticular scaffold formation and HEV or lymphoid vessel formation. In addition, positive feedback loops between LTi and LTo cells sustain sufficient activation signals for the development of SLOs. The location of PPs is fixed embryonically, and their formation cannot be induced by bacteria after birth, while CP and ILF formation is more flexible; the initiation of CPs or ILFs can occur both prenatally and postnatally. In addition, the unevenly colonized ILFs indicate that the gut microenvironment, which includes microbes and dietary metabolites, could exert a crucial role in ILF development.

ILC-driven TLSs are similar to secondary lymphoid organs (54–56). The formation of TLSs is under the continuous stimulation of ongoing chronic inflammation (54). Local activation of T cells and B cells in TLSs results in faster immune responses and better efficacy (57). LTi cells express integrin α4β7 that interacts with MAdCAM-1 on high endothelial venules (HEVs), which allows them to migrate toward lymph niches (future lymphoid organ sites) such as the intestine, fetal spleen and thymus (58–60); the migration of LTi cells leads to the expression of adhesion molecules and chemokines that are involved in lymphoid organogenesis through the lymphotoxin-α/β receptor (LTαR/LTβR) pathway (15, 61, 62). In addition, TNF expressed by LTi cells binds to TNFR1 on LTo cells and can facilitate SLO formation synergistically with the LTβR/LTαR pathway, activate the noncanonical NF-κB pathway to produce chemokines and adhesion molecules and enhance LTβR engagement, thereby forming a positive feedback loop (63). Consequently, LTi cells stimulate lymphoid tissue organizer (LTo) cells to secrete CXCL13, CCL19, and CCL21 through the LTβR pathway and consequently recruit hematopoietic cells via the expression of CXCR5 and IL-7R (64). The activated LTo cells could differentiate into fibroblastic cells, marginal reticular cells and follicular DCs, providing a reticular structure for migrated cells. In addition, activated LTo cells in turn recruit LTi cells via the expression of CXCL12, CXCL13, CCL19 and CCL21. The crosstalk between LTo and LTi cells and the increased LTα1β2 expression induced by B cells and LTi cells via CXCR5 signals ensures sustained LTo-LTi stimulation, which forms positive feedback loops (64, 65). Those recruited T cells, B cells, DC cells, etc., then form T or B-cell areas. Growth factors released by LTo cells, such as VEGF, FGF and HGF, contribute to the formation of HEVs and lymphatic vessels, which consequently ensures mature SLO formation (54) (**Figure 2**).

In mouse intestines, secondary lymphoid organ CPs, approximately 1000 cell clusters, are mainly composed of LTi-like cells, DCs. Stromal cell populations are situated at the bottom of intestinal crypt structures (21, 58). In addition, as a site where B cells are recruited, CPs can develop into ILFs, which resemble TLSs in structure (66), and accumulate more lymphocytes and stromal cells. Consequently, highly organized ILFs develop into mature lymphoid structures after bacterial colonization in the gut (58). LTi cells located in lymphoid niches trigger LTo cells to secrete CXCL13, CCL19 and CCL21 to recruit B cells, T cells and DCs to form ILFs via LTi cell-stromal gathering (16, 58, 67). The number and size of CPs and ILFs are variable, depending on the microbiome (68), while the number and size of location PPs are fixed embryonically. Moreover, the development of ILFs partially depends on Notch signaling induced by AHR, but when Notch signaling is lost, LTi cells, CPs and ILFs are less impaired than AHR deficits, indicating that there are different signaling pathways that have similar functions to Notch (69). However, LTi cells in mature mice failed to initiate the formation of SLOs; the specific regulatory mechanism is still unclear, but current studies suggest that these may be related to changes in biomarkers on LTi cells during different life stages.

Oxysterols are ligands for LTi-expressed G-protein-coupled receptor 183 (GPR183), which has been verified to regulate ILC3s since GPR183 can modulate immune cell migration (70). GPR183- LTi cells cause formation deficiency in CPs and ILFs, and in the Ch25h-lacking mouse model, the same phenotype was also observed. 7a,25-OHC (GPR183 ligand) produced by fibroblastic stromal cells attracts GPR183+ LTi cells to CP formation sites. This process stimulates the crosstalk of LTα1β2+ ILC3s and LTβR+ stromal cells, which facilitates GPR183+ B-cell recruitment to form ILFs (71). When lacking the GPR183 pathway, microbiota, CXCL13 and CCL20 could activate B-cell recruitment to form B-cell follicles and ILFs in the small intestine (72). Thus, the 7a,25-OHC-GPR183 pathway is crucial for lymphoid tissue formation in the colon, but this pathway is only an alternative in the small intestine (71–73).

LTi-induced TLS formation is linked with better survival and better prognosis. Consistently, a recent study found that in NSCLC, NCR+ ILC3s, similar to LTi cells, could induce the expression of VCAM-1 and ICAM-1 on MSCs and lead to the formation of lymphoid tissue (74). In addition, LTi cells or LTi-like ILCs also promote the formation of TLSs in extraintestinal tissues. B cells experienced more active effector differentiation, clonal proliferation and isotype switching, and T cells also expressed more activation markers in TLSs. Increasing evidence has shown that LTi-drived TLSs contribute to a favorable prognosis in extraintestinal cancers, such as lung cancer, pancreatic cancer and melanoma (54, 75). Thus, inducing the formation of TLSs may be a promising strategy for both intestinal and extraintestinal tumors.

## 5 Roles of different subtypes of ILC3s in maintaining intestinal homeostasis

ILC3s mainly reside in intestinal mucous, participate in innate responses and protect against pathogens (2). ILC3s are a large RORγt+ ILC population that can be divided into the following three distinct lineages: LTi cells, NCR+ ILC3s and NCR- ILC3s (30). NCR+ ILC3s can produce IL-17, IL-22 and GM-CSF, and mouse Nkp46+ ILC3s also express IFN-γ (76). The pathogen defense role of ILC3s predominantly relies on IL-22, and this function can be enhanced by RA (3, 19). NCR- ILC3s primarily express IL-17, while NCR+ ILC3s mainly produce IL-22. In the presence of IL-1β plus IL-23, mouse NCR- ILC3s could develop into NCR+ ILC3s in vitro (30). LTi cells facilitate the formation of lymphoid tissue at the fetal stage. It is difficult to identify LTi cells and NCR+ ILC3s based on biomarkers, but they have different development paths. In the adult stage, LTi-like cells are similar to LTi cells in gene expression, but they cannot initiate secondary lymphoid organ formation (7).

### 5.1 ILC3s

ILC3s play an important role in protecting against bacteria through the secretion of IL-22. GPR183, an ILC3-expressed chemotactic receptor, modulates the accumulation of ILC3s, which is required for the production of IL-22 (69, 77). Chu et al. confirmed that IL-22-producing ILC3s were reduced in GPR183-/- mice, suggesting that the GPR183 pathway promoted IL-22 production by inducing the accumulation of IL-22-producing ILC3s, and no other ILC3 subsets (77). IL-22 can induce the release of antimicrobial peptides (AMPs), establishing a gradient of bactericidal activity (78), facilitating antimicrobial defense and maintaining the epithelial barrier by promoting epithelial cell proliferation (79–81). Additionally, IL-22 has been proven to alleviate gut inflammation by promoting the production of mucus and then improving the epithelial barrier (82). It was observed that IL-22-deficient mouse models infected with Citrobacter rodentium developed UC (8, 19, 83). In addition, ILC3-derived IL-22 supports Lgr5+ intestinal stem cells (ISLs) to protect against inflammation (84). IL-22 acts directly on Lgr5+ ISLs, inducing phosphorylation of STAT3, which is vital for IL-22-dependent epithelial regeneration, especially after tissue damage (85). Moreover, IL-22-promoting fucosylation of intestinal carbohydrates favors commensal bacteria colonization but not pathogenic bacteria colonization because most of them cannot utilize carbohydrate fucose as an energy resource (86). Thus, IL-22 could prevent intestinal inflammation by promoting beneficial commensal bacterial colonization (43).

ILC3-derived IL-17 and GM-CSF also mediate pathogen defense, but their efficacy is not as potent as that of IL-22. NCR- ILC3-derived IL-17 expressed ROS and α-defensin to recruit neutrophils, thereby enhancing the epithelial barrier (30). GM-CSF can induce the generation of IL-10 and RA, inducing the formation of oral tolerance via activation of DCs and macrophages (30). Therefore, NCR+ ILC3s could protect homeostasis in the intestine through the efficacy of cytokines they secrete. IL-22 is a key ILC3-derived cytokine that protects the integrity of the epithelial barrier, regulates the gut microbiota and defends against pathogens. However, ILC3s also play an important role in extraintestinal host defense. Similar to ILC3 protection against intestinal bacteria, ILC3s also induce immune defense in lung diseases, especially Mycobacterium tuberculosis. Upon host infection with M.tuberculosis, ILC3s upregulate proinflammatory-expressing genes and thereby recruit macrophages and neutrophils to fight against the infection (87).

### 5.2 LTi/LTi-like cells

Both NCR+ ILC3s and LTi cells could produce IL-22 but in different niches of intestinal lymphoid structures/lamina propria. Deficiency of the ILC3 response results in expanding segmented filamentous bacteria (SFB). It increases the incidence of colitis (88–90). Nevertheless, the expansion of SFB and homeostasis dysbiosis were not observed in specific NCR+ ILC3-deficient mice, suggesting that LTi cells may have particular functions in ILC3-induced antimicrobial immunity (9). LTi cells, the first identified ILC subgroup, produce IL-22, IL-17A and IL-17F, and they develop from lymphatic tissue inducer precursors that differ from other ILCs (7). Although the lineage development of LTi cells is different from that of NCR+ ILC3s and all other ILC subtypes, both subtypes of ILC3s play an important role in mucosal protection (7, 12, 91). CCR6+ LTi cells locate in the intestine embryonically, develop distinctly from other ILC populations and, more importantly, promote lymphoid tissue development in the presence of lymphotoxin-β and TNF during embryogenesis (30, 71, 92).

CCR6+ LTi cells, the majority of ILC3s in lymphoid organs, internalize antigens and present antigens to CD4+ T cells, activating the production of T-cell-dependent antibodies (7). In the presence of IL-1β, LTi/LTi-like cells in lymphoid organs express CD80 and CD86 and produce IL-2, TNF-α and IFN-γ to fully activate T cells, and a specific discussion about the effects of LTi/LTi-like cells in antigen presentation and regulation of T cells will be presented in Section 5.1 (93).

In the fetal stage, LTi cells play a critical role in secondary organogenesis (94). As we mentioned above, LTo cells (95, 96), as a specialized stromal cell group, produce CXCL13, which recruits LTi cells to form the initial hematopoietic cell cluster (65, 95, 96).

## 6 ILC3s interaction with adaptive immune cells

### 6.1 T-cell

ILC3s could convert to MHC-II+ ILC3s via Basic leucine zipper ATF-like transcription factor (BATF)-induced enhanced chromatin accessibility of MHC-II antigen processing and presentation genes (97). MHC-II-expressing ILC3s present antigens to CD4+ T cells and result in suppression of cellular immunity and humoral immunity in the intestine (98). It has been demonstrated that MHC-II+ CCR6+ ILC3s could downregulate CD4+ T-cell abundance and TH17 cells while upregulating regulatory T cells (Tregs) through antigen presentation in the intestine (99). MHC-II+ CCR6+ ILC3-induced antigen presentation could result in the upregulation of Nur77 and Bim, which have been verified to be associated with negative selection in the thymus (99). Under infection with SFB and H. hepaticus, TH17 cells were expanded, and the differentiation of Tregs was impaired significantly in an MHC-II-deficient mouse model (100). This indicates that MHC-II+ ILC3s are necessary to protect the intestinal microenvironment by suppressing inflammatory T-cell expansion.

However, splenic ILC3s upregulate the expression of surface MHC-II molecules, activate CD4+ T cells and upregulate the expression of the costimulatory molecules CD80 and CD86 under stimulation with IL-1β (101). It has also been reported that the abundance of TH17 cells and IgG titers are significantly reduced in MHC-II-deficient ILC3 mouse spleens (93). These inconsistent results also showed that the function of ILC3s is associated with different microenvironments. In addition, the microbiota triggers the production of IL-23 and consequently downregulates MHC-II+ ILC3s under steady conditions, while virus-induced IFN-γ can promote the expression of MHC-II and induce the proliferation of CD4+ T cells under viral infection (101). Thus, alteration of the microenvironment could be an effector that regulates the MHC-II+ ILC3-driven T-cell response.

IL-2 has been reported to exert protective effects that could play a critical role in the generation and function of CD4+ T cells. Recently, Zhou et al. found that the transcription level of IL-2 in ILC3s is much higher than that in IL-2-producing CD4+ T cells through RNA sequencing of the small intestine. IL-2 transcription in ILC3s could be specifically induced by macrophage-derived IL-1β. Consistent with a mouse model, patients with Crohn’s disease have diminished IL-2+ ILC3s in the intestine but no remarkable difference in other IL-2-producing cells compared to healthy controls (102). However, MHC-II+ CCR6+ ILC3s could induce IL-2 withdrawal through their combination with IL-2 and initiate the TCR-induced apoptotic program, as the IL-2 requirement of T cells is intrinsic, but T-cell apoptosis could be reduced when given additional IL-2 (99). These results showed that a deficit in the IL-1β-ILC3-IL-2 axis could lead to changes in the abundance of CD4+ T cells and Tregs and impaired immune regulation in the intestine. Moreover, LTi-like cells induce T-memory and T-independent antibody responses by expressing APRIL, BAFF, CD30L and OX40L (88). Memory CD4+ T cells are RORγt dependent. A marrow chimeric mouse model showed that LTi cells are crucial RORγt-expressing cells, supporting memory CD4+ T-cell survival in the absence of antigen stimulation (88).

### 6.2 B-cell

ILC3s could mediate IgA responses with or without interacting with T cells. Human tonsillar NKp44p ILC3s secrete the B-cell activation factor BAFF, indicating that ILC3s support B-cell activation and survival in mucosal tissues to facilitate the production of IgA antibodies (89). In addition, CCR6+ LTi cells induce secondary lymphoid structure formation and contribute to the accumulation of B cells, which is required to synthesize T-independent IgA (90). Within PPs and ILFs generated by LTi cells, B cells can also interact with CD40L+ CD4+ T cells and then convert into IgA+ plasma cells, promoting intestinal immunity (66, 90). Strikingly, one recent study proved that ILC3s could suppress the generation of IgA+ B cells to protect against both commensal and pathogenic bacteria by presenting antigens to T follicular helper cells (Tfhs) and suppressing the Tfh response, except in PPs or the intestinal lamina propria (98). Tfh cells, a distinct lineage of CD4+ T cells, play an important role in assisting B-cell responses (103). In mice that lack ILC3-intrinsic MHC-II expression (MHCIIΔILC3), the Tfh response increased in mesenteric lymph nodes, and IgA+ B cells notably increased in the colon. A number of studies have reported that mucosal bacteria such as Helicobacter and Mucispirillum help to build up mucosal defense in the early stages of life (104). However, mucosal bacteria fail to colonize colon niches due to dysregulation of the IgA response in the absence of ILC3s (98). Thus, ILC3s could mediate intestinal bacterial colonization to maintain homeostasis by regulating the IgA response. Therefore, further studies examining the connections between LTi cells and B-cell immunity for the important role of LTi cells in adaptive immunity may be helpful for the treatment of cancer, autoimmune diseases and inflammatory diseases.

## 7 Crosstalk between ILC3s, dietary metabolites and the gut microbiota alters the intestinal microenvironment

Trillions of microbes colonize the intestine; the crosstalk between commensal microbes and ILC3s residing in the gut plays a vital role in regulating the intestinal microenvironment and intestinal health (105). Current studies have found that dietary metabolites can indirectly modulate the intestinal microenvironment by altering the gut microbiota. In this review, we present the idea that the diet could mediate the intestinal microenvironment through crosstalk between the gut microbiota and ILC3s. Dietary metabolites impact the structure and activity of intestinal microbes and then regulate intestinal immunity. Recent studies have proposed that dietary metabolites modulate the functions of ILC3s through TFs and thereby stimulate specific transcription programs (71). A ketogenic diet, characterized by low carbohydrate and high fat, could alleviate colitis by reducing ILC3s by altering the gut microbiome. In addition, a ketogenic diet increased the abundance of intestinal bacteria such as Akkermansia and butyric acid-producing Roseburia, which are conducive to the maintenance of intestinal health. It has been confirmed that the microbiota plays an important role in the physiology of ILC3s and that a ketogenic diet-altered intestinal microbiota alleviates inflammation by reducing the frequency of ILC3s (106). Consistently, the endogenous Trp catabolite kynurenine can also modulate ILC3s by activating the intestinal microbiota (27). Kynurenine activates the AHR-Notch pathway and consequently promotes ILC3 production of IL-22 (107). ILC3-regulated immune responses allow colonization of commensal microbiota while providing resistance to C. albicans. These results suggest that dietary metabolites could activate the ILC3-induced mucosal innate response, which mainly relied on IL-22, with the participation of gut microbiota. However, it is still unclear which signals from the microbiota specifically induce IL-22 production.

Vitamin A, an important nutrient, is enriched in fruits, vegetables, dairy products and so on. The metabolites of vitamin A, RA, play a vital role in fetal LTi cell development and lymphoid tissue formation (108). ID2+ RORγt+ CD4+ LTi cells differentiated from ID2+ RORγt+ CD4- LTi cells initiate the formation of SLOs (109). Blockade of the RA signal resulted in a decrease in ID2+ RORγt+ CD4- LTi cells and SLO density, while the frequency of ID2+ RORγt+ CD4+ LTi cells increased after RA stimulation of lymph node cells (109). In addition, a diet lacking RA hinders ILC3 proliferation and the development of a secondary lymphoid organ (110). Moreover, mice fed a vitamin A-deprived diet had a notable decrease in ILC3s and ILC3-derived cytokines IL-22 and IL-17, while ILC2s and ILC2-derived cytokines such as IL-4, IL-5 and IL-13 were increased, suggesting that vitamin A may be related to the equilibrium between ILC2s and ILC3s (111). Additionally, RA secreted by DCs can accelerate the differentiation of ILC1s into ILC3s (30, 39).

Short-chain fatty acids (SCFAs), metabolites of dietary fibers (DF), are mainly produced by microbial fermentation (112). As the most abundant microbial metabolites in the intestine, SCFAs, combined with G-protein-coupled receptors (GPCRs), could support antibody production, promote T-cell production of IL-10 and stimulate ILC2s and ILC3s (105). It has been observed that the abundance of ILC3s in mice fed high DF (high SCFAs) was much higher than that in mice fed low DF (high SCFAs), while ILC1s in mice fed high DF were much higher; the expression of IL-17A, IL-22 and Ffar2 (GPCR expressed by ILCs) in mice fed low DF was obviously lower than that in mice fed high DF (113). SCFAs activate the STAT3, STAT5, mTOR and PI3K pathways to support ILC proliferation following Ffar2 activation. In addition, Chun et al. found that Ffar2 could also regulate the expression of ILC3 apoptotic or survival factors (105). Notable decreases in gut pathogens and inflammation remission in the high DF group showed the positive role of SCFAs in improving enteric immunity against intestinal infection. Taken together, these results show that dietary metabolites could modulate ILC3-induced intestinal responses through the activation of ILC3s or the regulation of ILC3 conversion. In addition, as we mentioned above, 7a,25-OHC binding to GPR183 could also contribute to host defense by supporting SLO formation (71).

**Figure 3:**
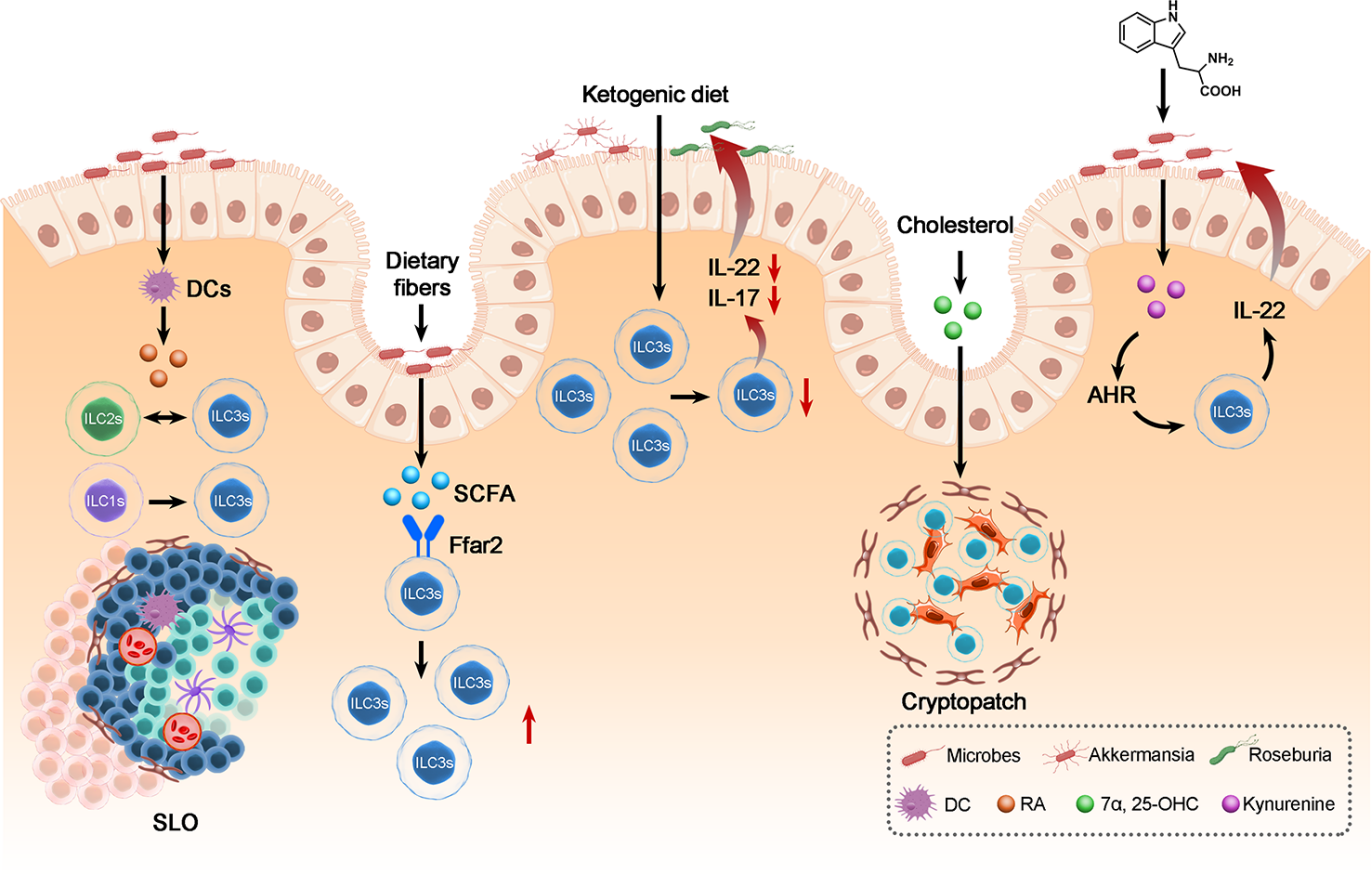
The crosstalk between ILC3s, dietary metabolites and the gut microbiota in maintenance of intestinal homeostasis. Dietary metabolites derived from microbes can regulate the biology of ILC3s: Including modulates the frequency of ILC3s, the conversion between ILC3 and ILC1 or ILC2 and the development of SLO, thereby affecting the balance of gut immunity and thus the biology of gut microbes and the homeostasis of intestine.

Considering the crosstalk between dietary metabolites that exerts an eminent effect on ILC3s, understanding the mechanism of crosstalk between enteric microbes and ILC3s through diet metabolism helps to alleviate the progression of intestinal inflammatory diseases (**Figure 3**).

## 8 ILC3s in intestinal disease: Foes or friends?

### 8.1 Dysbiosis of ILC3s contributes to the development of IBD

IBD, characterized by chronical gut inflammation, mainly consists of ulcerative colitis (UC) and Crohn’s disease (CD) (114). Recent studies found that ILC3s support the mucosal homeostasis of intestine but the dysregulation of ILC3s population would result in the formation and progression of IBD (30). Increased IFN-γ-producing ILC1s were also found in inflammatory intestine from IBD patients (19). It has been proven that in gut inflammatory tissue, the frequency of ILC3s was decreased in patients with IBD, but the number of ILC1s was increased, which suggests that the conversion of ILC3s to ILC1s could also contribute to the deterioration of IBD (115). Moreover, the reduction in ILC3s are also closely linked with the severity of IBD (115). The increased frequency of ILC2s is also related to the progression of IBD (19, 30). ILC2s upregulate the plasticity toward ILC1s and ILC2s separated from mucosa of CD patients also showed the IFN-γ producing capability, which significantly contributes to intestinal inflammation. In addition, ILC2-derived IL-33 was found to be increased in colitis mice model. IL-33 therapy and ILC2 removal was identified to be helpful for inflammation alleviation (116).

The GPR183 pathway is closely related to IBD (69). Colonic inflammation could activate GPR183 pathway by increasing 7a,25-OHC production. In CD40-AB treated mice model, the amount of 7a,25-OHC in colon would increase, in response to inflammation (71). Overexpressed 7a,25-OHC excessively activates GPR183, promoting ILC3s migration toward 7a,25-OHC and thus contributing to ILC3 population dysregulation which further induces IL-22 overexpression (69). ILC3-derived IL-22 can help to maintain the homeostasis in intestine but overexpression of IL-22 would recruit excessive neutrophil cells to produce proinflammatory cytokines and thus lead to quick and swift enhancement of epithelial barrier permeability (30). However, DCs stimulated by IL-25 downregulate IL-22 production, suggesting a complicated regulatory mechanism of the DCs-ILC3 axis (11).

Vonarbourg et al. found that NKR+ RORγt+ LTi cells release IL-22 to activate the epithelial barrier, but NKR+ RORγt- LTi cells (ex-ILC1s) could produce IFN-γ, which can induce the progression of colitis (117–119). Therefore, the RORγt gradient could mediate the functions of NKR+ RORγt+ LTi cells. T-bet and RORγt act reversely and negatively regulate each other (120, 121). Chronic colitis triggers the release of IL-12, which promotes NKR+ RORγt+ ILC3 conversion into IFN-γ-producing ILC1s. Accumulation of ILC1s in inflammatory tissue and overexpression of IFN-γ results in epithelial barrier damage and aggravation of intestinal inflammation (40).

### 8.2 Promising immunotherapy in IBD treatment

Current medical treatment of IBD mainly focused on anti-inflammation drugs. The usage of them could alleviate inflammation but cause a variety of side effects. Therefore, it is urgent to study new drugs with better safety and higher efficiency. Over the past decade, ILC3s have been recognized to highly correlate with IBD pathogenesis, and thus drugs targeting ILC3-regulation factors may provide a novel strategy for IBD treatment (122).

TNF could result in intestinal epithelium cell death and chronic inflammation in intestine. Blocking of TNF is widely used in IBD treatment. Recent studies have found that ILC3s could protect intestinal epithelium from TNF-related apoptosis through production of heparin-binding epidermal growth factor–like growth factor (HB-EGF). IL-1β induces ILC3 to produce PGE2. Then, PGE2 significantly stimulates ILC3s to produce HB-EGF. The abundance of HB-EGF+ ILC3s was found to be decreased in inflammatory intestine tissue (123).

Hueber et al. found that the IL-17A inhibitor, secukinumab, showed worse efficacy than placebo. CD persistent disease activity was observed in patients treated with secukinumab, leading to inflammation aggravation and severe adverse events in patients with apparent inflammation (124). Compared with the satisfying efficacy of secukinumab in psoriasis, unpleasant outcomes in CD treatment may be related to different immune microenvironment in these diseases. IL-17A participates as an important factor in innate intestinal protection (19); therefore, inhibition of IL-17A may leads to homeostasis dysbiosis (20). Clinical remission can be observed in patients with CD who were treated with ustekinumab, an antibody of the shared p40 unit of IL-12 and IL-23 (30, 125). Ustekinumab treatment partially restored the balance of ILC subsets with a decrease in ILC1s and an increase in ILC3s (126). However, p40 antibody only attenuates CD in the first two month since ustekinumab not only inhibits IL-12-induced ILC3-ILC1 conversion but also suppresses IL-23-induced IL-22 production, blocking the crucial anti-inflammation responses (127). Thus, an antibody, specifically blocking IL-12 may show better efficacy in IBD treatment than an anti-p40 antibody.

Unlike the favorable prognosis in CRC, the increased TLS in the DSS-colitis mouse model is linked with inflammation progression. Previous studies have demonstrated that in response to dysregulation of intestinal commensal bacteria and immune tolerance loss, TLS develops during chronic inflammation. TLS formation is an important characteristic of UC, which is more likely to develop into extraintestinal inflammation than CD (128). Unlike the protective role in CAC, it has been found that intestinal TLSs can produce antibodies aberrantly under immune dysregulation and lead to IBD progression. Thus, site-directed delivery of LTβR inhibitors may be a more viable modality for the treatment of IBD with less harm to other lymphoid structures. However, how to utilize the antimicrobial defense function of TLSs and avoid dysbiosis-related disease progression and the detailed regulatory mechanism of TLSs in IBD remain unclear.

As we mentioned above, MHC-II+ ILC3s could downregulate the frequency of CD4+ T cells and other inflammatory cells to maintain intestinal homeostasis. Recent studies have observed that MHC-II expression was obviously reduced on ILC3s from pediatric patients with IBD. Thus, MHC-II+ may be a promising target for IBD treatment considering their crucial role in inducing apoptosis of CD4+ T cells (99).

Considering the complicated regulatory mechanism of intestinal immune and biological functions of cytokines in IBD pathogenesis, it is hard to choose appropriate inhibition therapy in CD treatment. Hence, further study of ILC3s mechanisms in IBD is necessary.

### 8.3 Roles of ILC3s in colorectal cancer

IBD is likely to develop to CRC since IBD often results in chronic inflammation in gut mucosa (19). ILC3s was identified as possessing both pro- and antitumor properties (129). The frequency of ILC3s decreased while that of ILC1s increased in CRC tissue compared to benign adjacent tissue, which is consistent with IBD. CCR6+ LTi cells could restrict the TH17 response and gut inflammation via MHC-II to limit the invasion and progression of CRC (130). However, the level of CCR6+ MHC-II+ LTi cells was lower in CRC tissue (131). The ratio of CD4+ T cells to MHC-II-expressing LTi cells notably increased in CRC, which indicates that the interaction between T cells and CCR6+ MHC-II+ LTi cells in CRC tissue is interrupted due to the decrease in MHC-II+ LTi cells (131). Consistently, transforming growth factor-β (TGF-β) in the tumor microenvironment suppressed the upregulation of HLA-DR, CD80 and CD86, and consequently inhibited antigen-presentation, leading to diminished T-cell responses (132).

In contrast, many studies identified that ILC3-specfic IL-22 contribute to progression and metastasis of intestinal tumors (19). In a bacteria-driven CRC mice model, ILC3s and IL-22 are closely linked with the progression of metastatic CRC (133). Soluble IL-22 binding protein derived from DCs can neutralized IL-22 and then suppress cancer progression by preventing the binding between IL-22 and IL-22R (134). Huber et al. found that in an IBD mouse model, IL-22BP-/- mice were more likely to develop CRC (135). IL-22BP is highly expressed in the normal microenvironment and downregulated when intestinal tissue are damaged (135). IL-22 could promote gut tissue repair and epithelial cell proliferation during intestinal damage, but uncontrolled IL-22 production would result in tumorigenesis (136).

Microbial dysbiosis contributes to the pathogenesis of CRC through regulation of ILC3s (137). Previous studies confirmed that the accumulation of IL-22 was postnatal. IL-22 frequency gradually increased after birth. Moreover, it has been shown that the complexity of the intestinal microbiome is consistent with IL-22 production (138). C. albicans could stimulate macrophage IL-7 production, elicited ILC3s to produce IL-22, and consequently promote CRC formation. IL-7 combined with IL-23 and IL-1β has a synergistic effect on IL-22 secretion and leads to CRC progression (138, 139). However, IL-1β could also support the capacity of ILC3s to produce CXCL10 and high expression of CXCL10 is associated with better antitumor responses (129). These results may suggest that the controversial role of cytokines may be attributed to the difference in the intensity of signaling and the tumor microenvironment.

### 8.4 Novelty perspective on treatments of colorectal cancer

Similar to IBD, the excessive expression of IL-22 and dysregulation of ILC3s also contribute to the development of CRC. It has been confirmed that treatments targeting ILC3 regulating factors also have encouraging efficacy in patients with CRC. LTi-driven TLS was closely linked with favorable prognosis in CRC patients (140). Consistently, high TLS was identified as a sensitive marker of more prolonged survival in clinical trails (141). It has been confirmed that ILFs recruited lymphocytes to the tumor microenvironment (TME), facilitating the antitumor immune responses (73). Vaccination treatment using engineered CCL21-expressing DCs increase the formation of TLS in melanoma through the recruitment of T cells. The crosstalk of immune cells sited in TLS enhanced the antitumor response and contributed to the regression (142).

Moreover, recent studies has reported that under the stimulation of IL-1β, ILC3-like cells upregulated the expression of MHC-II (HLA-DA), CD70, CD80 and CD89, while TGF-β could suppress this process and the expression levels of MHC-II and CD89 were comparable to that in professional antigen-presenting cells (APC). Enhanced antigen-presenting function could facilitate cytomegalovirus specific memory CD4+ T cells. These results suggest ILC3-like cells could respond to specific cytokines, increase antigen-presenting properties and consequently regulate memory CD4+ T-cell responses. Thus, whether we could enhance CD4+ T cells response in CAC treatment by using cytokines that mediate the antigen-presenting role of ILC3-like cells may be a new perspective for CAC treatment (132). Thus, we suggest that treatment using engineered chemokine delivery cells that activate TLS in CRC and facilitating antigen-presenting to prompt T-cell responses in tumor milieu may contribute to the treatment of CRC.

## 9 Conclusion

While many regulatory mechanisms of Group 3 ILCs in maintaining intestinal homeostasis and promoting or alleviating autoimmune diseases have been identified, several transcriptional pathways and functions of specific cytokines are still unclear. Previous studies have reported the following findings: 1. dysregulation and conversion of ILC3s could accelerate the progression of tumor and autoimmune disease while ILC3s supports the immunity against pathogens and help maintain gut homeostasis. 2. ILC3s play a protective role by producing cytokines, inducing TLS formation and supporting the adaptive immune response. 3. Autoimmune diseases or tumors account for ILC3s decrease and the conversion of ILC3s to other ILCs subsets; this could also lead to disease progression and hence positive-feedback loops. 4. The crosstalk between ILC3s and microbes plays crucial role in the immune environment *via* diet metabolites produced by microbes. Here, we point to 5 critical questions about how to utilize ILC3s in controlling autoimmune diseases and tumors. 1. Could we block the expression of specific TF like T-bet to restrict ILC3s conversion to ILC2s or ILC1s to alleviate disease progression? 2. What signals or pathways ensure that LTi cells support SLO in the fetal stage and could we induce SLO formation and enhance the intestine’s immune system? 3. Could we promote anti-pathogen immunity through a specific diet? Further understanding of ILC3s regulatory mechanism and the crosstalk of ILC3s and other ILC subsets, adaptive immune cells, lymphoid tissues and microbes will have a place in future inflammatory disease and tumor treatment. 4. Can all ILC conversion be reversed and how can ILC plasticity be regulated to alleviate inflammation or tumor progression? 5. Why does TLS exert a different role in IBD and cancer, and what is the detailed regulatory mechanism of IgA production and host defense functions in TLS? Thus, further study of ILC regulatory mechanism may help to understand the roles of ILC in cancer and inflammatory diseases and provide a novel perspective for innovative and targeted therapies.

## Author contributions

YZ: literature search, study selection, draw figure, manuscript writing. ZM:literature search, manuscript writing, revision of manuscript. PR: study design, revision of manuscript. XF, JW, HT and JL: revision of manuscript. All authors contributed to the article and approved the submitted version.
